# Activation of AQP2 water channels by protein kinase A: therapeutic strategies for congenital nephrogenic diabetes insipidus

**DOI:** 10.1007/s10157-021-02108-6

**Published:** 2021-07-05

**Authors:** Fumiaki Ando

**Affiliations:** Department of Nephrology, Tokyo Medical and Dental University, 1-5-45 Yushima, Bunkyo-ku, Tokyo, 113-8510 Japan

**Keywords:** AQP2, Congenital NDI, PKA, AKAPs

## Abstract

**Background:**

Congenital nephrogenic diabetes insipidus (NDI) is primarily caused by loss-of-function mutations in the vasopressin type 2 receptor (V2R). Renal unresponsiveness to the antidiuretic hormone vasopressin impairs aquaporin-2 (AQP2) water channel activity and water reabsorption from urine, resulting in polyuria. Currently available symptomatic treatments inadequately reduce patients’ excessive amounts of urine excretion, threatening their quality of life. In the past 25 years, vasopressin/cyclic adenosine monophosphate (cAMP)/protein kinase A (PKA) has been believed to be the most important signaling pathway for AQP2 activation. Although cAMP production without vasopressin is the reasonable therapeutic strategy for congenital NDI caused by V2R mutations, the efficacy of candidate drugs on AQP2 activation is far less than that of vasopressin.

**Results:**

Intracellular distribution and activity of PKA are largely controlled by its scaffold proteins, A-kinase anchoring proteins (AKAPs). Dissociating the binding of AKAPs and PKA significantly increased PKA activity in the renal collecting ducts and activated AQP2 phosphorylation and trafficking. Remarkably, the AKAPs–PKA disruptor FMP-API-1 increased transcellular water permeability in isolated renal collecting ducts to the same extent as vasopressin. Moreover, derivatives of FMP-API-1 possessed much more high potency. FMP-API-1/27 is the first low-molecular-weight compound to be discovered that can phosphorylate AQP2 more effectively than preexisting drug candidates.

**Conclusion:**

AKAP-PKA disruptors are a promising therapeutic target for congenital NDI. In this article, we shall discuss the pathophysiological roles of PKA and novel strategies to activate PKA in renal collecting ducts.

## Introduction

Aquaporin-2 (AQP2) water channels present in renal collecting ducts play an essential role in body water homeostasis. AQP2 was cloned in 1993, which contributed significantly toward elucidating the urinary concentrating mechanism [1]. Over time, due to advancements in analytical techniques of molecular biology, it has been understood that the signaling pathways for AQP2 activation are far more complicated than anticipated. To obtain further clarification of the mechanisms underlying AQP2 regulation, several researchers began investigating the pathophysiology of congenital nephrogenic diabetes insipidus (NDI).

Congenital NDI is characterized by increased excretion of diluted urine despite appropriate secretion of the antidiuretic hormone vasopressin. In severe cases, patients’ urine volume reaches up to 10–20 L/day [2]. Excessive urine output and thirst cause severe reductions in the quality of life; however, only symptomatic treatment approaches are currently available [3]. Although thiazide diuretics together with indomethacin or the potassium-sparing diuretic amiloride is helpful in reducing the degree of polyuria in patients with congenital NDI, this treatment strategy does not completely eliminate polyuria. A reduction of only 50% of total urine excretion was observed with regular medication therapy [4]. Consequently, patients experience complications, such as renal dysfunction, growth disorder, and mental retardation. Therefore, development of curative therapies for treating patients with congenital NDI is required. This has created a strong driving force for elucidating the various regulatory mechanisms of AQP2. A detailed understanding of pathophysiological and molecular mechanisms of AQP2 could contribute to generating a novel therapeutic strategy for congenital NDI.

**Fig. 1:**
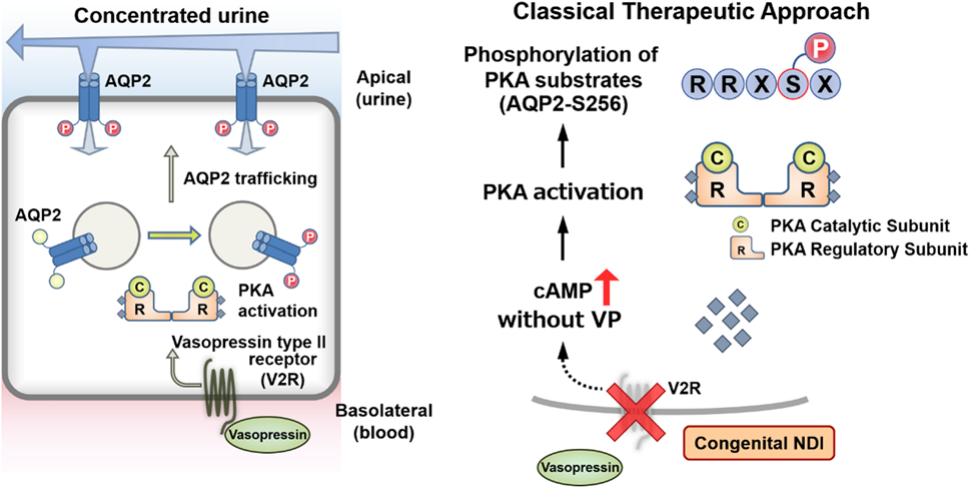
Classical therapeutic strategy for congenital NDI. (Left) Binding of vasopressin to V2R in the basolateral membrane stimulates adenylyl cyclase and then activates the cAMP/PKA signaling pathway. PKA then phosphorylates AQP2, resulting in the translocation of cytosolic AQP2 to the apical plasma membrane. Water is reabsorbed from urine through AQP2 water channels, and urine is concentrated. (Right) Mutations in V2R account for 90% of all diagnosed congenital NDI cases. Elevation of intracellular cAMP levels by bypassing defective V2R achieves AQP2 activation without vasopressin

In approximately 90% of patients with congenital NDI, the underlying cause is loss-of-function mutations in the vasopressin type 2 receptor (V2R) [5]. Defective V2R prevents elevation of intracellular cyclic adenosine monophosphate (cAMP) levels in response to vasopressin. On this basis, cAMP production without vasopressin has long been considered as a promising therapeutic target for congenital NDI (Fig. 1). cAMP-elevating agents, such as G protein-coupled receptor (GPCR) agonists and phosphodiesterase (PDE) inhibitors, have been intensively explored as a treatment option [6]. In addition to the elevation of cAMP levels, several strategies have been advocated, such as rescuing mutant V2R with chemical chaperones, activation of calcium signaling, and increasing intracellular cGMP levels [7, 8]. These strategies and preexisting drug candidates have definitely activated AQP2 phosphorylation and trafficking in vitro, but unfortunately, they failed to sufficiently increase urine concentration in vivo. Consequently, no specific pharmacological drugs have yet reached clinical application. Different approaches are required to more potently activate AQP2. In this review, I focus on cAMP-dependent protein kinase (PKA) as a promising therapeutic target for congenital NDI.

## Molecular mechanisms of AQP2 activation by vasopressin

In response to dehydration, the antidiuretic hormone vasopressin is secreted from the posterior pituitary. Vasopressin binds to V2R in renal collecting ducts and increases the intracellular concentration of cAMP. cAMP-induced trafficking of AQP2 to the apical plasma membrane is critical for the reabsorption of water from urine to improve dehydrated states in the body. The most important factor determining the intracellular localization of AQP2 is AQP2 phosphorylation. AQP2 is a protein made up of 271 amino acids, and the phosphorylation sites responsible for AQP2 trafficking are accumulated at the C-terminus of AQP2. The phosphorylation status of serine 256 (S256), S261, and S269 in particular has been commonly referred to as a useful marker for AQP2 activity [9, 10].

From the mid-1990s to the mid-2010s, functional analyses of these AQP2 phosphorylation sites were conducted using serine to alanine (A) or serine to aspartate (D) phospho-mutants. S256 is considered a “master regulator” of AQP2 activity because AQP2-S256A is constitutively localized in intracellular vesicles, whereas AQP2-S256D is retained at the apical plasma membrane [11, 12]. Moreover, PKA is believed to be responsible for AQP2 phosphorylation at S256 for two reasons. The first is that PKA is directly activated by the vasopressin/cAMP signaling pathway. PKA is a tetramer comprising two regulatory (PKA R) and two catalytic (PKAc) subunits in its inactive form. The binding of cAMP to each PKA R subunit results in the dissociation of PKAc from the PKA R subunits and subsequent phosphorylation of target sequence RRXS/T by the PKAc subunit. The second reason is that AQP2-S256 contains a PKA phosphorylation consensus sequence, RRXS^256^.

In addition to AQP2(S-D) or (S-A) phospho-mutants, AQP2 phosphorylation had been evaluated by autoradiography, however, the dynamic changes in AQP2 phosphorylation at S256, S261, and S269 were not accurately detectable. Phospho-specific antibodies provided new insights into AQP2 phosphorylation. Unexpectedly, AQP2 phosphorylation at S256 was found to be constitutively high irrespective of vasopressin stimulation [13]. By contrast, vasopressin significantly decreases AQP2 phosphorylation at S261 and increases it at S269. Importantly, these changes in AQP2 phosphorylation at S261 and S269 show good correlation with the accumulation of AQP2 at the apical plasma membrane, and therefore, S261 and S269 are often used as markers of AQP2 activity [8].

## Importance of PKA in AQP2 regulation

Although the AQP2 phosphorylation status at S261 and S269 highly reflects AQP2 activity, PKA does not phosphorylate these sites directly [14], causing uncertainty regarding the importance of PKA in the mid-2010s. The widespread use of the CRISPR-Cas9 gene-editing technology has solved this controversial issue. PKA-knockout cell lines of renal cortical collecting ducts (mpkCCD cells), which exhibit endogenous AQP2 expression [15, 16], provided an advanced experimental system to explore the precise role of PKA in AQP2 regulation.

Isobe et al. used CRISPR-Cas9 system to delete both PKAc α and β subunits to completely eliminate PKA activation in response to cAMP [17]. Interestingly, AQP2 mRNA and protein expressions were undetectable in the PKA-knockout mpkCCD cell lines. AQP2 overexpression revealed that the vasopressin-induced AQP2 dephosphorylation at S261 and AQP2 phosphorylation at S269 were impaired, resulting in unresponsiveness of AQP2 trafficking to the apical plasma membrane. These data indicated that PKA is the most important kinase regulating AQP2 expression, phosphorylation, and trafficking.

The downstream targets of PKA that mediate PKA-induced AQP2-S261 dephosphorylation and AQP2-S269 phosphorylation remain unknown. Quantitative phosphoproteome analysis of mpkCCD cell lines in the presence or absence of vasopressin revealed that six protein kinases (Sik2, Cdk18, Camkk2, Prkd2, Mapk3, and Mylk) were involved in the vasopressin/cAMP/PKA signaling pathway [18]. In addition to kinases, phosphatases in the vasopressin signaling pathway contribute to AQP2 phosphorylation [19]. Although the omics approach provides comprehensive information, PKA substrates critical for AQP2 phosphorylation remain unidentified. A culprit PKA substrate that directly phosphorylates AQP2-S261 and AQP2-S269 may be a potential therapeutic target for congenital NDI.

## A-kinase anchoring proteins (AKAPs) regulate AQP2 activity

Although PKA is a ubiquitously expressed protein kinase, it is sequestered at specific subcellular locations, thus leading to compartmentalized cAMP/PKA signaling [20]. The intracellular localization and activity of PKA are determined by its scaffold proteins, which are known as AKAPs. AKAPs bind to both PKA R subunits and other signaling molecules, thereby allowing the phosphorylation of PKA substrates specifically [21]. AKAPs spatially and temporally regulate the PKA signaling networks.

In renal collecting ducts, AKAPs are involved in AQP2 phosphorylation. It has been reported that AKAP220 colocalized with AQP2 in the cytosol of the inner medullary collecting ducts and increased forskolin-induced AQP2 phosphorylation [22]. AKAP18δ is located on AQP2-bearing vesicles and directly interacts with PDE4D and PKA [23]. PDE4D phosphorylated by PKA accumulates together with AQP2 at the plasma membrane, where it is involved in terminating cAMP-dependent water reabsorption. In addition to AKAP220 and AKAP18δ, 43 AKAP genes and more than 70 functionally distinct AKAP proteins have been identified [24]. Recently derived omics data indicate that several AKAPs are expressed in renal collecting ducts [25–27]. They probably coordinate PKA activity in renal collecting ducts to regulate AQP2 phosphorylation in the vasopressin signaling pathway.

Disruption of the AKAP–PKA interaction is another approach to regulate AQP2 activity. In this regard, Ht31 is the best-characterized peptide disruptor comprising a PKA-anchoring domain of a human thyroid AKAP [28]. Ht31 and AKAPs competitively bind to the PKA RII subunits and induce the dissociation between AKAPs and the PKA RII subunits. Ht31 has been traditionally used to inhibit local PKA activity by displacing PKA from AKAPs–PKA–PKA substrates complexes, whereas Ht31 increased PKA activity in baby hamster kidney cells [29]. In mpkCCD cell lines, we also observed that Ht31 activated PKA, resulting in AQP2 phosphorylation and trafficking.

**Fig. 2:**
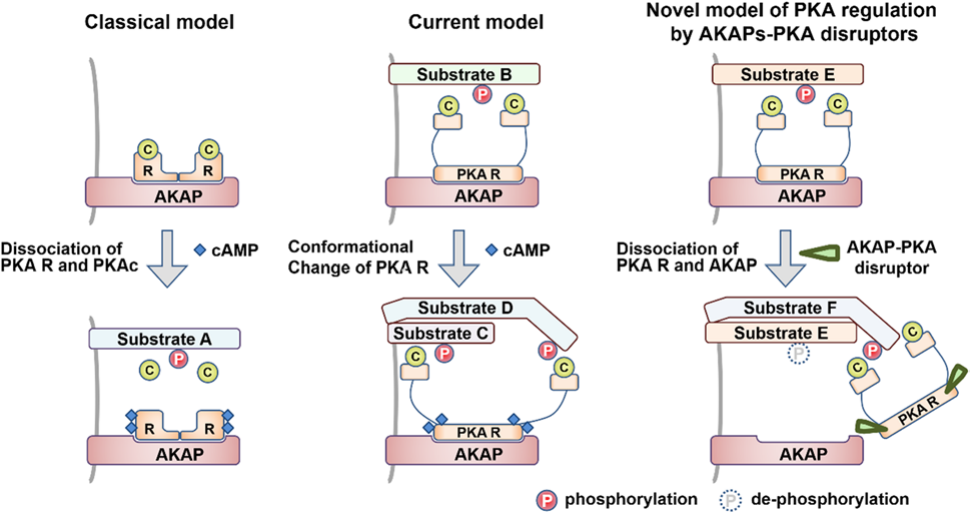
Paradigm shift of PKA regulatory mechanisms. (Left) Elevation of cAMP triggers the release of the PKAc subunits from the PKA R subunits in the classical model. AKAP is important for compartmentalization of PKA activity in cells. (Mid) Catalytically active PKA holoenzyme remains intact within AKAP assemblies [30]. Dissociation of PKAc subunits is not required for PKA activation. (Right) Unanchored intact PKA holoenzyme by AKAP–PKA disruptors changes the phosphorylation status of PKA substrates

This finding can be explained by the paradigm shift of PKA regulatory mechanisms (Fig. 2). The classical model of cAMP-induced PKA activation is characterized by the dissociation of active PKAc subunits from PKA R subunits. Smith et al. found that assemblies of AKAP–PKA R subunits–PKAc subunits remain intact, although local cAMP production stimulates the kinase activity of PKA [30]. The anchored PKA holoenzyme action is much more restricted than originally anticipated. These results suggested that unanchored intact PKA holoenzyme by Ht31 still possesses kinase activity and enhances phosphorylation of PKA substrates in mpkCCD cell lines.

## FMP-API-1 activates AQP2 in vivo

Unfortunately, owing to size and chemistry, the bioavailability and renal drug delivery of the Ht31 peptide inhibitor are insufficient in vivo. We next focused on a low-molecular-weight compound, FMP-API-1, which binds to the allosteric site of PKA R subunits and inhibits AKAPs–PKA interactions [31]. Similar to Ht31, FMP-API-1 increased the activity of PKA and AQP2 in mpkCCD cell lines [32]. Furthermore, FMP-API-1 increased the water permeability in isolated renal collecting ducts to the same extent as vasopressin. In contrast to vasopressin, FMP-API-1 did not increase AQP2 mRNA and protein expression in mpkCCD cell lines.

**Fig. 3:**
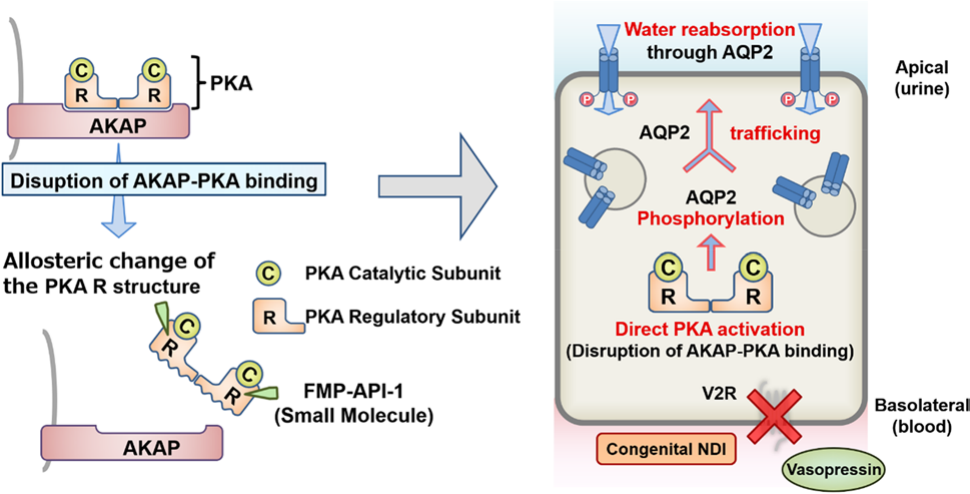
FMP-API-1 activates AQP2 in vivo. FMP-API-1 binds to an allosteric site in the PKA R subunits that causes disruption of AKAP–PKA binding [31]. In renal collecting ducts, unanchored PKA activates AQP2 independently of vasopressin and cAMP. AKAP–PKA disruptors are novel therapeutic targets for congenital NDI caused by V2R mutations

AKAP–PKA disruptors are a promising therapeutic target for congenital NDI (Fig. 3). However, FMP-API-1 has poor solubility and decreases the effect of AQP2 activation in vivo. We then synthesized derivatives of FMP-API-1, such as FMP-API-1/27. FMP-API-1/27 exhibited a high potency and strongly changed the AQP2 phosphorylation at S261 and S269 to the same extent as vasopressin in mouse kidneys [32]. FMP-API-1/27 successfully increased the urine concentrating ability in a V2R-inhibited NDI mouse model.

## Importance of drug-target profiling of FMP-API-1/27

We had previously investigated the effects of rolipram, a PDE4 inhibitor, and Wnt5a, an activator of calcium signaling, on AQP2 [8, 33], but we found that both failed to sufficiently increase the urine concentrating ability in vivo. FMP-API-1/27 possesses high potency compared with that of preexisting drug candidates. Although FMP-API-1/27 disrupts the binding between AKAP18δ and PKA R subunits [31], its precise target in renal collecting ducts remains to be identified. Currently, there is a lack of availability of AKAP-knockout mice with impaired urine concentrating ability. AKAP18-knockout mice exhibit no obvious phenotype of polyuria or syndrome of inappropriate antidiuretic hormone secretion [34]. Moreover, the vasopressin/cAMP/PKA system of AKAP220-knockout mice is intact, and their urine is normally concentrated in response to dehydration [35]. Drug-target profiling of FMP-API-1/27 could be useful to determine the most important AKAP responsible for AQP2 activation.

## Conclusion

PKA in renal collecting ducts is directly activated by AKAP–PKA disruptors. PKA is also known to be involved in a variety of pathologic states other than congenital NDI. cAMP modulators, such as PDE inhibitors, GPCR agonists, and GPCR antagonists, are used in daily clinical practice. AKAP–PKA disruptors have the potential to be developed into a completely new class of therapeutic agents. PKA RIIβ subunit–knockout mice exhibit antiaging phenotypes with enhanced tolerance to obesity and diabetes mellitus [36, 37]. Such effects can also be anticipated from AKAP–PKA disruptor administration.
